# Incorporating textual network improves Chinese stock market analysis

**DOI:** 10.1038/s41598-020-77823-3

**Published:** 2020-12-01

**Authors:** Yi Li, Zichuan Mi, Wenjun Jing

**Affiliations:** School of Statistics, Shanxi University of Finance and Economics, Taiyuan, Shanxi China

**Keywords:** Statistics, Applied mathematics, Computer science

## Abstract

This study adopts the textual network to describe the coordination among the interplay of words, where nodes represent words and nodes are connected if the corresponding words have co-occurrence pattern across documents. To study stock movements, we further proposed the sparse laplacian shrinkage logistic model (SLS_L) which can properly take into account the network connectivity structure. By using this approach, we investigated the relationship between Shenwan index and analysts' research reports. The securities analysts’ research reports are crawled by a famous financial website in China: EastMoney, and are then parsed into time-series textual data. The empirical results show that the proposed SLS_L model outperforms alternatives including Lasso-Logistics (L_L) and MCP-Logistic (MCP_L) models by having better prediction performance. Besides, we search published literature and find the identified keywords with more lucid interpretations. Our study unveils some interesting findings that the efficient use of textual network is important to improve the predictive power as well as the semantic interpretability in stock market analysis.

## Introduction

In the financial markets, the exponential increase in the amount and types of data available to investors prompted some companies to completely change their business strategy and adopt a Big Data investment framework. With the advent of social networking sites, like Facebook, Blog, Twitter, etc., the latest information about public opinions has become abundant. In fact, investigating stock market by using online financial textual data has attracted increasing attention in academic and real-life business^1–3^. Some early empirical research in behavioral finance argued that investment decisions are subject to the effect of investor sentiment^4,5^. Such inference was further confirmed by the studies of Li^6^ and Schumaker et al.^7^. The researchers discovered that emotion from online social media can be explored for the stock market trend, e.g. whether the stock price is going up or down according to the news media affects investor confidence with a delay of at least one month^8^. However, when encountering the sheer volume of online resources on various websites, users find it very difficult to distinguish between posts containing facts, rumours, guesses, and fake news. The effects of imperfect information make investors unable to attract the stock market reactions accurately.

Nowadays, increasing numbers of studies quantitatively examine the information percolation of research reports on stocks^8,9^. One of the earliest studies, by Previts et al.^10^, argued that the analyst reports are of great importance to investors' information needs. Asquith, Mikhail, and Au^11^ then discovered that the argument of the downgrade reports has a positive relationship with the stock market reaction. Twedt and Rees^12^ further investigated the effect of reports, finding that stocks could be positively correlated with the textual tone of the reports. Hence, extracting valuable information from analysts' research reports is considered to be a more stable and trustworthy source and is likely to enrich the knowledge of investors and affects their trading activities.

The Chinese language consists of several thousand characters known as *Hanzi*, with a word consisting of one or more characters. Compared to English, Chinese is more complicated in terms of recognition, segmentation and analysis^13^: Chinese does not have an explicit word boundary marker and do not contain whitespace between words; Chinese words are not clearly marked grammatically; Chinese also contains a very large number of homophones in sentences^14^. Hence, the amount of research on Chinese social media analysis is limited and the number of research articles on using the textual analysis results to predict the stock market in China is even lower. Fortunately, more researchers have started to conduct Chinese textual analysis in the recent years. For instance, Wang et al.^15^ deploy text mining and statistical model to predict stock market price movement using Weibo mood. In other words, the increasing expansion of Chinese internet market has fuelled a growing important research field on text mining in Chinese.

This paper investigates stock market movement (whether the price will be up or down) that can be deemed a classification problem. The fundamental idea of our multi-variable classification method is that related vectors of numeric feature values originate from words of documents in stock prediction. The classic logistic regression model has long been regarded as allowing highly accurate prediction as well with feature interpretability of a probabilistic nature. However, there are some challenges when performing logistic regression model on text mining. The first challenge is “curse of dimensionality” due to the massive amounts of words. Generally, a document collection contains thousands of words, and a bag of words representation of a document will probably have a very high dimensionality. Furthermore, a sparse matrix is created in order to indicate which document each word occurs in. The matrix is sparse because most words occur just in a few documents. A large number of penalized regression approaches are proposed, including sparse penalty and thereby breaking the curse of dimensionality to improve model interpretability^16–20^. Such proposals are the least absolute shrinkage and selection operator (LASSO), elastic net, the smoothly clipped absolute deviation (SCAD), the minimax concave penalty (MCP) and others. Among these methods, MCP has been demonstrated the preferred performance on the selection of predictors and computational efficiency^21,22^.

The second challenge is the insufficiently independent words as the individual unit of text mining, which have usually been used to express the semantics of the document. The pioneering study shows that the text's semantics exist inherent interplay of words, analyses their interconnections, distinguishes "signal from noise" and provides a more comprehensive description of the document^1^. For example, if the size of a laptop is described as a word "thin", then it is considered as a positive thing, whereas if the sentence contains "thin" relative to the weight of an individual then it is considered as a negative statement. By uncovering these latent relations between words, the meanings of words can be derived. From the perspective of each single relationship and then extend that perspective to the whole text network, each word represents a node. Links between nodes denote that the corresponding words are semantically "correlated". This network structure can be done by one individual who can provide personal meaning to the events, or meaning can also be provided at the supra-individual level. In the latter case, words with a high connectivity are more likely to be involved in an important semantic process. Hence, semantic relations between words can be constructed for whole text network analysis. Incorporation the latent structure information has been proved to significantly improve predictive power. Li et al.^23^ proposed a network-constrained regularization method and found that it facilitates the selection of predictors using the network information. However, as far as we known, none of the previous network-based analyses have been trained on the background of text predictive procedures, which is the focus of this paper.

The main goal of this work is to combine the text network in forecasting stock market and thus be more informative. To this end, we study network-constrained sparse logistic regression, which has exploited a new perspective for the research and application of text mining. Firstly, we have constructed the textual co-occurrence network, and studied its properties. Then the composite penalization is presented. This method is built upon a combination of the minimax concave penalty and the Laplacian penalty in order to achieve the properties of sparsity and smoothness^24^. In particular, Laplacian penalty takes full advantage of the existing network information to smooth the differences between coefficients of tightly connected words, aiming at improving prediction performance. Our work fills the gap in incorporating textual network into forecasting stock market, and undergirds the important conclusion that with some delay word patterns in security analysts' research reports affect the weekly Shenwan Stock closing index of the real estate sector. Besides, we offer a number of advantages over alternative approaches to improve the prediction performance.

The remainder of this paper is organized as follows. “Methodology” introduces the novel textual predictive approach. In “Empirical research”, we review the overall process on stock movement forecasting and the empirical research. Next section reviews the “Experimental results”. Finally, this paper is summarized and discussed in “Conclusions”

## Methodology

Details about the designed textual predictive approach are given in this section. Suppose that the stock market movement is $Y = \left\{ {y_{1} , \ldots ,y_{t} , \ldots ,y_{n} } \right\}$, where $y_{t}$ denotes an observation of categorical response variable at time $t$, which indicates the fluctuation direction in terms of increase ($\uparrow$) or decrease ($\downarrow$). There exists a market predictor set ${\text{T}} = \left\{ {T_{1} , \ldots ,T_{t} , \ldots ,T_{n} } \right\}$ where $T_{t}$ represents the textual data available at time $t$, and $n$ is the size of observations. The term $x_{i,t}$ extracted from the text $T_{t}$ is the frequency count. Thus, the textual data $T_{t}$ is defined as a word vector ${\text{X}}_{t} = \left\{ {x_{1,t} , \ldots ,x_{i,t} , \ldots ,x_{m,t} } \right\}$, where $m$ is the number of vocabularies.

One assumption here is that the text $X_{t - 1}$ could deliver underlying information about the future market and the ability to forecast stock movement ${\text{y}}_{t}$. Assuming that ${\text{y}}_{t}$ follows a binomial distribution, a binomial logit model is defined by

$$ \pi_{t} = \frac{{\exp \left( {X_{t - 1}^{^{\prime}} \beta } \right)}}{{1 + \exp \left( {X_{t - 1}^{^{\prime}} \beta } \right)}} $$

where $\beta$ is the vector of regression coefficients, whose length is the vocabulary size $m$. Therefore, the objective function of logistic regression can be written as

$$ {\text{L}}\left( {\beta ;y_{t} ,X_{t - 1} } \right) = y_{t} \left( {X_{t - 1}^{^{\prime}} \beta } \right) - \log \left( {1 + e^{{X_{t - 1}^{^{\prime}} \beta }} } \right) $$

### Construction of text networks

In text network analysis, a vertex corresponds to a word. Links between words can be regarded as meaningful information in sentences. Furthermore, our study assumes that the meanings of words embodied in a text could be modelled as a network of linked words. That is, this type of information about nodes, along with the links between them, should be used for uncovering, understanding, and exploiting the semantics of text as a whole.

Consider an undirected co-occurrence network represented by a weighted graph $${\mathcal{G}} = \left( {{\text{V}},{ }{\mathcal{E}},{\text{W}}} \right)$$ with vertex set $${\text{V}} = \left\{ {1, \ldots ,{ }m} \right\}$$ corresponding to $$m$$ keywords, edge set $${\mathcal{E}} = \left\{ {\left( {i,j} \right):\left( {i,j} \right) \in {\text{V}} \times {\text{V}}} \right\}$$, the degree set $${\text{D}} = {\text{diag}}\left( {{\text{d}}_{1} ,{\text{d}}_{2} , \ldots ,{\text{d}}_{m} } \right)$$, where $${\text{d}}_{m}$$ is the degree of vertex $$m$$, and the set of weights $${\text{W}} = \left\{ {{\upalpha }_{i,j} :\left( {i,j} \right) \in {\mathcal{E}}} \right\}$$. Here $${\upalpha }_{{{\text{i}},{\text{j}}}}$$ is the weight of edge (*i, j*), which measures the strength of connection between keyword vectors $$i $$ and $$j$$, with 1 for complete-link and 0 for no-link, defined by

$$ \alpha_{i,j} = I\{ \left| {\hat{r}_{i,j} } \right| > r\} $$

where $\hat{r}_{i,j}$ is the Pearson correlation coefficient, and $r$ is the threshold parameter based on the $p$-value for determining the significance between keyword vectors $i$ and $j$. There are other quantitative measurements of defining the correlation methods, including the Euclidean distance, Spearman’s correlation, power adjacency function, and so on (Huang et al. 2011). In this paper, we only consider the Pearson correlation. Finally, we construct the adjacency matrix A = (${\upalpha }_{i,j} , 1 \le i,j \le m$), which is used for the Laplacian penalty related to the network, as we illustrate below.

### Sparse Laplacian shrinkage

The regression coefficients ${\upbeta } = \left\{ {{\upbeta }_{1} , \ldots ,{\upbeta }_{m} } \right\}$ can capture the effects of the term variable $x_{i,t}$. In addition, the term network's depicting relationships between predictors are informative. The penalized method for network-constrained sparse logistic regression is defined by

$$ \hat{\beta } = \arg \min \left\{ {\frac{1}{n}\mathop \sum \limits_{i = 1}^{n} \left\{ { - L\left( {\beta ;{\text{y}},{\text{x}}} \right)} \right\} + {\text{P}}_{\lambda ,\gamma } \left( \beta \right)} \right\} $$

where  

$${\text{P}}_{\lambda ,\gamma } \left( {\upbeta } \right) = \mathop \sum \limits_{i = 1}^{m} \rho (\left| {{\upbeta }_{i} } \right|;{\uplambda }_{1} ,{\upgamma }+ \uplambda _{2} \upbeta ^{\prime}L\upbeta. $$

Here ${\text{P}}_{\lambda ,\gamma } \left( {\upbeta } \right)$ is a sparsity and smoothness based penalty function with emphasis on the underlying network structure ${\mathcal{G}}$. Two tuning parameters $({\uplambda }_{1} ,{\uplambda }_{2} )$ with ${\lambda}_{1} \ge 0$ and ${\lambda}_{2} \ge 0$ control the degree of regularization, $\rho$ is the minimax concave penalty with two regulation parameters $\left( {\uplambda _{1} ,\upgamma } \right)$.

For sparsity penalty, we use the minimax concave penalty in the first penalty term, defined as

$$ \rho \left( {\left| {{\upbeta }_{i} } \right|;\lambda_{1} ,{\upgamma }} \right) = \lambda_{1} \mathop \sum \limits_{j = 1}^{m} \mathop \smallint \limits_{0}^{{\left| {{\upbeta }_{j} } \right|}} \left( {1 - \frac{x}{{\gamma {\uplambda }_{1} }}} \right)_{ + } dx $$

In this analysis, terms are supposed to be the semantic units for selection. Hence, the first penalty promotes sparsity and directs the search to more meaningful item combinations.

For network penalty, the Laplacian penalty is adopted in the second penalty term, defined as $\lambda_{2} \beta^{\prime}L\beta$. The Laplacian matrix $L$ is always positive semi-definite and defined with respect to the network ${\mathcal{G}}$, with L = D–A, which satisfies

$$  \upbeta ^{\prime}L\upbeta  = \sum\limits_{{1 \le j \le k \le m}} {a_{{ij}} } \left( {\upbeta _{j}  - \upbeta _{k} } \right)^{2}   $$

It accommodates the network structure. For instance, items with higher connectivity are considered to have closely related semantic units. We adopt a constraint on the contrast between ${\upbeta }_{j}$ and ${\upbeta }_{k}$ to improve the smoothness of estimated coefficients with respect to the prior structure information and thus results in more interpretable identification of the items.

The prediction procedure follows the stock movement prediction method described in the work of Bergmeir and Benitez^25^. In particular, the SLS method has three tuning parameters: (λ_1_, λ_2_ and γ). The parameter λ_1_ controls the level of sparsity, λ_2_ controls the degree of the coefficient smoothing, and the third parameter ${\upgamma }$ in addition to λ_1_, governs the concavity of the sparsity penalty function. In practice, we search in a grid of λ_1_, λ_2_ values with λ_2_ ∈ (0, 0.001, 0.01, 0.1, 1, 10) and choose the values of λ_1_, λ_2_ that maximize accuracy of predictions. The third parameter γ controls the concavity of the MCP penalty. When $\gamma \to \infty$, MCP reduces to the L_1_ penalty. In practice, we fix ${\upgamma }$ to the default value of 2.7 to reduce computation, and the prediction accuracy is usually not sensitive to ${\upgamma }$ values.

To be more specific, we adopt V-fold cross-validation to choose the optimal combination of λ_1_ and λ_2_, and then partition the dataset into separate training and test data. Furthermore, variable selection and parameter estimation are accomplished on the training data with cross-validation to choose the tuning parameters, and the test data is used to assess the forecasting abilities. The accuracy of predictions is defined as the area under the curve (AUC), which can be defined as the probability that a randomly chosen positive instance is ranked higher than a randomly chosen negative instance. A test with AUC > 0.9 has high classification accuracy. Moreover, it is easy to compare with uncertainty forecasting, such as the Random Walk Hypothesis, because the random classification model could cause an AUC value of 50%^26,27^.

## Empirical research

### Experimental design

**Figure 1:**
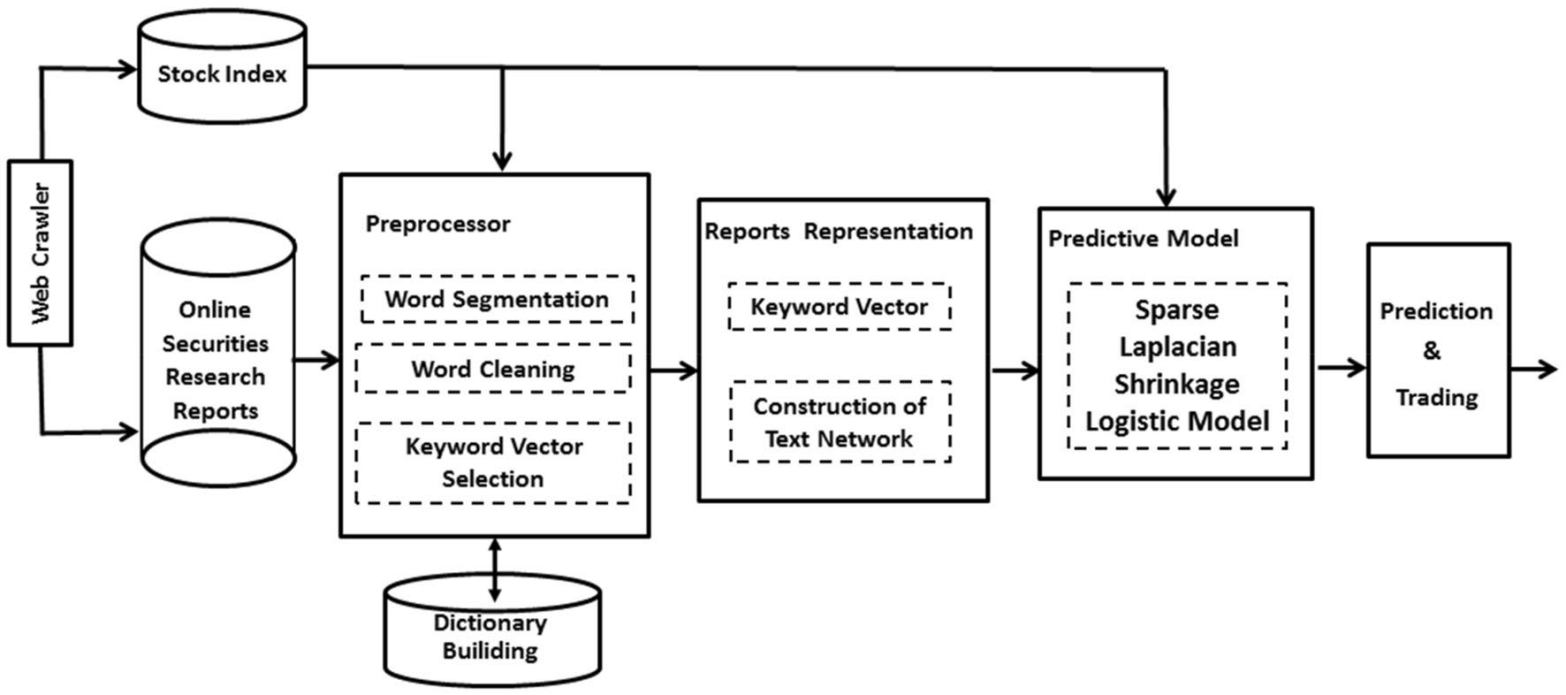
The flowchart of the text mining processing.

Our paper relates to research that the power of information on securities analysts' research reports and applies an SLS_L model to predict the stock market reaction. The flow of our method has some of the components depicted in Fig. 1. Our overall processor provides a solid basis to determine the internal connections between securities analysts' online research reports and stock price movements. At one end textual data obtained from online sources and stock index are fed as input to the system and at the other end, some stock predictive movements are generated as output. The first step is data collection using the web crawler, and then extracting relevant information from a suitable dataset. Words and phrases that signal a stock movement are important and should be extracted. Thus, the preprocessor is a crucial part of text mining and is mainly composed of the following common preparatory steps: dictionary building, word segmentation, and word cleaning. After data cleaning, we measure the time difference between each word and target indicator, and then choose the list of words that indicate the most significant correlation, in a sense that the subset is as small as possible but still retains all the relevant information.

In statistical terminology, a word is an observed variable, and a document is a list of observed variables representing an instance. Clearly, the list of words used the text representation are retrieved from the message corpus based on actual occurrences of the words. Building on the network construction, we examine important network properties such as density, centrality and K-core. At the subsequent step, we predict market movement direction using sparse group Laplacian shrinkage logistic model and assign weights based on text network information to keywords in proportion to the market movement. The remaining keywords serve as a determinate variable in our prediction model. Each step will be elaborated on Fig. 1.

### Data descriptions

For the stock market, the weekly Stock closing index of the real estate sector from the Shenwan Research database (SWS, http://www.swsresearch.com/EN/) has been collected ranging from May 14, 2015 to September 18, 2017^28,29^. The overall data includes 108 trading weeks. The sample of stocks being studied are 136 firms that together made up the weekly SWS index of real estate sector. The prediction target in this paper is the performance of the stock movement. The Stock closing index of the real estate sector is labelled suitably using a simple method. If the stock index at time $$t$$ is larger than that at time $$ t - 1$$, the direction at time $$t$$ is 1, otherwise, direction at time $$t$$ is 0, that is, the stock movement performance is mapped into 0 or 1 as the prediction target.

The textual data are collected by crawling and parsing large amounts of web pages about the most popular and prestigious financial website in China, EastMoney (http://www.eastmoney.com/), by searching the content of all securities analysts’ research reports in the China real estate sector from 14 May 2015 to 18 Sep. 2017. The online reports are to give a complete and timely description of stock information from the security company, which will dynamically capture the stock trend, hence assist the user to make an investment decision^30,31^. We crawl down and compile from the Internet using Python libraries, which are composed of two major libraries: the target URL list generating library (Requests) and the HTML page parsing library (Beautiful Soup).

For each research report in the China real estate sector on the site, the script recorded the date of the posting, the title, the security company name and the body of the message. The text of a report includes varying levels of topics, such as the company's significant recent developments, industry dynamics, a critique of the company's management and board, and investment risk, etc. There are a total of 2082 reports from 65 security companies. Each research report is timed to the day, the mean security reports in a day are 3.8 and the number of words in a report is most frequently between 500 and 2000. In order to assess the content of the research report, we employ well-established text mining, which is executed to transform raw documents into lists of keyword vectors and adjacent matrix of the network.

### Predictive keyword vector extraction

The research report is employed as our textual source, mainly because these texts might expose some things that firms may not like to tell their outside listeners in blunt terms. By implementing this action, the raw text of a report published in the same week is merged into one document, where larger reports have adequate data distribution within a week's span. After eliminating noise and outliers, our research reports are narrowed down to 108 windows over the 28 months and the mean security reports in a window are 17, with a maximum of 36. Each window is converted into the bag of words, represented as a vector of counts. The following steps are conducted on a single document. Our judgment and cleaning criterion is elaborated on as follow.

#### Step 1: dictionary building

The Sogou Pinyin input method is a dominant input software in China, and Sogou cell lexicon can be obtained from the Sogou Pinyin input method official website (https://pinyin.sogou.com/dict/). These lexica come from the analysis of millions of Chinese web pages generated by the Sogou search engine^32^. Therefore, we choose the Sogou cell lexicon as the dictionary and exclude the uncommonly used words. Then we own the remaining 63,320 words or phrases as the external dictionary, which is embedded in step 2 in order to decrease ambiguities.

#### Step 2: Text segmentation

Unlike English where text processing starts with tokenization, Chinese text segmentation is the procedure of identifying the boundaries between semantic units, for instance, phrases or words. In this step, we use the Jieba package to perform text segmentation^33^. This segmentation approach is mainly based on a Hidden Markov model, and adding our own custom dictionary obtained from step 1 can ensure a higher rate of correct segmentation. To discern words, the Viterbi algorithm used in this package is to find the maximum tangential points based on the word frequency^34^.

#### Step 3: words cleaning

Since stop words do not contribute to the analysis in textual data without dependency on a particular topic, we remove these words with the same roots appearing in the stop-list such as numbers, more white spaces, tabs, punctuation characters, stop words, etc. In addition, we build stop-list that contains 75 non-semantic words such as "是(is)", "的(of)", "关于(about)", "如何(how)", and so on. We remove a word with a proportion in all documents was smaller than 80% because the chi-squares statistics is known not to be reliable for the low-frequency terms.

Using the above step 1–step 3, an initial set of 3285 keywords was built.

#### Step 4: keyword vector selection

There are thousands of words in documents. If we choose all words as features, it will be impossible to do forecasting since the computer cannot process such enormous amounts of data. Thus, we need to choose the most meaningful and representative units for prediction. There are very popular selection methods, for instance, chi-square statistics^35^, information gain, mutual information, document frequency and latent semantic analysis^36^. In this paper, we use chi-square ($\chi^{2}$) independent statistics to test the frequency count of each item in each report data and the market indicator fluctuations measured in terms of increase ($\uparrow$) or decrease ($\downarrow$) during each time period of one week. The statistic test is compared to the chi-square distribution with one degree of freedom. After the above filtering, 56 words remained at the 5% significance level in our trial. We concluded that the 56 words provide sufficient evidence to determine that there is an association between the nature of the frequency items and the market fluctuations. Therefore, we directly put the 56-word count vectors as the most predictive variable into the construction of the network and the predicted model.

## Experimental results

### Network results

**Figure 2:**
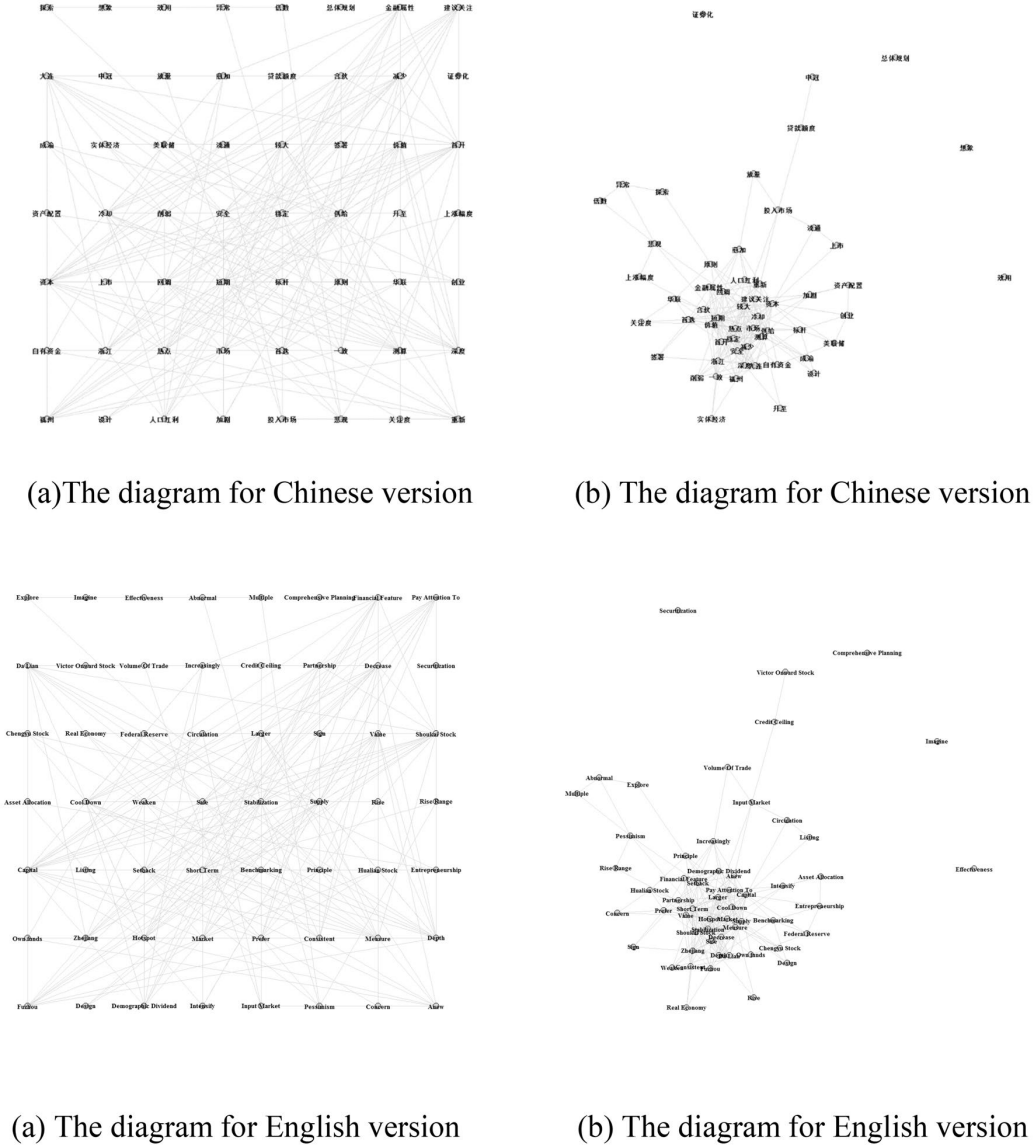
Keywords network. (**a**) The simple grid layout of a graph; (**b**) the node is scaled using the Fruchterman–Reingold layout algorithm.

Following Nassirtoussi et al.^1^, we refer to the 56 keywords as "representative textual features". Each keyword is a node in the network. A link between nodes indicates that co-occur within the two-keyword window with a statistically stronger frequency, which can effectively capture meaningful information^37^. Based on the keyword vector, a 56 × 56 undirected adjacency matrix is eventually constructed for network analysis. Figure 2a shows a simple grid layout of a graph which introduces the overview of the network on the basis of connectivity. Figure 2b shows the node is scaled using the Fruchterman–Reingold layout algorithm, which aims to keep adjacent vertices close to each other while ensuring that vertices are not too close to each other. Depending on the graph layout, we explore the characters of the text network. The graph demonstrates the prominent role and the comparative importance of keywords. More specifically, it can be seen that the keywords "Hotspot(热点)" and "Short Term(短期)" are central points of information flow. Keywords "Pessimistic (悲观)" and "Input Market (投入市场)" are supposed to be intermediary points. Other keywords seem to serve as peripheral points.

To do more detailed network analysis and achieve much more insightful and interesting ramifications, nevertheless, networks need to be distilled into key quantitative indicators that can be operationally defined and practically measured. We consider the following indicators to explore the cohesion, integration, composition and structure of the text network.

*Density* is a measure of network cohesion. In this sense, density represents the proportion of observed links in a network that are actually present. The value can be in the range from 0 to 1, where 0 indicates networks with no relationships and 1 indicates networks with all possible relationships. Our network density is 0.1695, meaning that there are 522 links in 56 × 56 adjacency matrix. Thus, the textual network is a sparse network. This result is significantly affected by the fact that text network of research reports on stocks is loos knit instead of densely connected.

*Centrality* is an indicator to find the most important keywords within a network. Betweenness is a centrality measure of a node within a network. Specifically, betweenness centrality measures the number of times a vertex acts as a link along the shortest path between two other nodes. A vertex with high betweenness has a high probability to control the flow of information in the network. In our study, only 44 vertices of the betweenness centrality are greater than zero. The word "Short Term" is the most influential keywords, with a betweenness centrality of 287.89, followed by "Pessimistic", "Input Market", "Larger" and "Market". The word "Short Term" takes up a position in the centre of the network and has a great effect on other words in the text network, which suggests that the factor like "Short Term" plays an important role in the interactivity of the stock market and the real estate market. This is because that our work focuses mainly on stock price reactions in the short term.

*K-core* is an indicator to identify closely interlinked subgroup within a network, which indicates a coalition of many keywords who have many stable patterns with each other. A K-core measure is used to find whether the network is structured. Table 1 shows that the cores become more interlinked when $k$ increases from zero, which indicates that our text network has a structural property rather than random^38^.

**Table 1:** Results of the K-core collapse sequence analysis.

k-core	0	1	2	3	4	5	6	7	8	9	10
k-remainder	5	3	7	3	5	3	3	5	3	1	18

### Prediction results

One simple hypothesis about the validity of a text network is that the text network is very noisy if the stock movement can be forecasted well using textual data only. In this instance, textual data seem to be sufficient to forecast. At the same time, integration of the network structure may not increase the accuracy of prediction much. On the contrary, if the prediction accuracy in the SLS_L model is improved, the use of network information could be more effective.

To test the above hypothesis, we conduct an evaluation of prediction performance using threefold cross-validation. For each fixed ${\uplambda }_{2}$, we can obtain the second tuning parameter besides ${\uplambda }_{2}$ by using the number of steps for the MCP optimal solution. The prediction results for the proposed Sparse Laplacian Shrinkage-Logistic (SLS_L) model based on 5 replicates are calculated as AUC = 0.9360, ${\uplambda }_{1}$ = 0.0251, ${\uplambda }_{2}$ = 0.001. Given these descriptive results, ${\uplambda }_{1}$ performs the variable selection and control the level of sparsity, and ${\uplambda }_{2}$ controls the degree of the coefficient smoothing, that is, the similarity between coefficients. When ${\uplambda }_{2}$ is around zero, the network structure is very noisy. In our study, the values of the tuning parameter ${\uplambda }_{2}$ are non-zero in all replicates. This illustrates the prediction accuracy of SLS_L model, which seems to perform better as it uses the network information.

To evaluate the effectiveness of network information from another point of view, Lasso-Logistics (L_L) and MCP-Logistic (MCP_L) models are applied to assess the prediction accuracy of the proposed SLS_L model. These comparisons of results demonstrate that the proposed SLS_L model (AUC = 0.9360) has the highest AUC in comparison with L_L(AUC = 0.8344) and MCP_L(AUC = 0.8707). The AUC values conclude that the proposed method can achieve lower prediction errors and higher prediction accuracy in the stock direction of change and outperforms L_L and MCP_L models in predicting the SWS week index of real estate sector. It provides evidence of the advantage of appending the network penalty and smoothing over the similarity between words.

In addition, the impact of the time lag on the market reaction is considered in this work. The up and down of the stock price is predicated on the subsequent $n$ weeks (five trading days). The results of the experiment show that in general, the AUC on the next one week was the highest, yielding up to 0.9360 for the SWS index of real estate sector. In the similar result, Asquith et al.^11^ discovered that analyst reports on companies can affect their market's reaction with five trade days delay. In the market, the trading date is the day that an investor's order is executed. Our result shows that some time is still needed for getting news to trading. It is reasonable to expect that public opinion on the market will only affect the stock fluctuation with some delay.

In our experiment, 56 words are shown to provide sufficient evidence to determine that there is an association between the nature of the frequency items and the market fluctuations. However, among these 56 words, the coefficients of 25 words shrink to zero when using all of the three models (MCP_L, SLS_L and L_L models), meaning that they are not effective. The remaining 31 words are effective, that is, the coefficients of them are not zero in at least one of the three models. In our manuscript, Table 2 shows the 31 words. It is expected that the evaluation of prediction performance can also provide an indirect evaluation of the textual implications of the models and representative features. To gain further insights, we now more closely investigate overlapping keywords detected by MCP_L, SLS_L and L_L models. These 25 keywords of overlaps are identified by all the models. Some keywords are expressed as the expectations or needs of investors and their trading activities, like pessimistic (悲观), Cool down (冷却), Rise (升至). Some keywords represent the current event and financial topics, like Demographic Dividend (人口红利), Real Economy (实体经济), Securitization(证券化). Some keywords indicate sufficient statistical meaning, that is, a stable relationship between keywords, like Intensify (加剧), Prefer (首先). Some keywords reflect correspondence to target indicator, like company name, city name. Searching published literature suggest that these keywords may have important implications. Recent evidences in behavior finance indicates that emotion influence stock market returns^6,39^. Previous studies suggest that the demographic variable can be related to the information component determining long-horizon stock market returns^40^. Westerhoff shows the interactions between the real economy and stock market^41^. Fontana and Godin find the links between housing market and financial sector by taking into account the securitization process^42^. Our result indicates events that most certainly influences stock market prices, public sentiment and opinion may play an equally important role in predicting stock movement.

**Table 2:** Results of the K-core collapse sequence analysis.

Keywords	Coefficient		
	MCP_L	SLS_L	L_L
Fuzhou(福州)	− 1.0156	− 0.3635	− 0.0823
Design(设计)	− 6.2383	− 0.9961	− 0.2950
Demographic dividend(人口红利)	− 1.4596	− 1.4300	− 0.7243
Intensify(加剧)	− 2.3491	− 0.8559	− 0.3031
Input market(入市)	− 1.4538	− 0.4034	− 0.1640
Pessimism(悲观)	6.6403	1.0625	0.3359
Concern(关注度)	− 10.1856	− 1.9920	− 0.8522
Anew(重新)	− 1.8171	− 0.3550	− 0.0789
Own funds(自有资金)	− 4.1016	− 0.7022	− 0.2374
Zhejiang(浙江)	0	− 0.2541	− 0.0970
Hotspot(热点)	− 0.2639	0	− 0.0019
Prefer(首选)	− 0.7123	− 0.2818	− 0.0752
Consistent(一致)	0	0	− 0.0087
Listing(上市)	− 0.3414	− 0.1252	− 0.0275
Principle(原则)	− 3.5691	0	0
Entrepreneurship(创业)	− 4.4760	− 0.9795	− 0.2328
Cool down(冷却)	− 4.3086	− 1.0358	− 0.0480
Rise(升至)	− 0.4476	− 0.4925	− 0.2201
Rise range(上涨幅度)	4.5206	0.9423	0.1197
Chengyu stock(成渝)	− 5.2876	− 1.9413	− 0.2541
Real economy(实体经济)	− 3.7955	− 1.0270	− 0.1717
Federal reserve(美联储)	− 1.9805	− 0.6351	0
Circulation(流通)	− 1.1935	− 0.5240	− 0.1364
Sign(签署)	− 1.3929	− 0.3504	− 0.0544
Volume of trade(放量)	− 2.0652	− 0.3127	− 0.0281
Securitization(证券化)	− 1.7006	− 0.4444	− 0.1080
Explore(探索)	0	0.4535	0
Imagine(想象)	7.2612	1.9061	0.4485
Effectiveness(效用)	6.5592	1.9769	0.4402
Multiple(倍数)	2.9660	1.1980	0.0678
Comprehensive planning(总体规划)	5.9179	0.9804	0.2849

The striking finding in Table 2 is that only five out of 25 terms have a positive impact on financial markets. Apparently even the positive word "Securitization(证券化)" received a negative connotation. The strongest positive indicator is "Imagine(想象)", and the strongest negative indicator is "Concern(关注度)". This finding seems to echo what Soroka^43^ and Wu et al.^44^ discovered earlier—responses to positive and negative information are asymmetric—that negative information has a much greater impact on individuals' attitudes than does positive information.

## Conclusions

This paper fills the gap in literature by integrating textual network in forecasting stock market and offers a number of advantages over alternative approaches. The sparse laplacian shrinkage logistic regression demonstrates state-of-the-art text predicting classification while producing sparsity and smoothness of efficient models. A richer penalty also may prove useful. Those used in our studies are not only informative in the statistical sense but also represent knowledge of the semantic network. We used text network analysis in order to take full advantage of semantic relations, whereas previous text mining mainly emphasized words as independent variables with no connections. The features of the semantic network structure provide significantly effectiveness of text mining.

Our experimental findings suggest textual information in the online analyst reports obtains better performance. Our work on stock-predictive text mining is specifically researched in a sector context, e.g. the web pages of all Chinese analysts' research reports of real estate sector from a well-known financial website. Furthermore, the textual information can give a full description of a specific stock or industry sector in order to help investors consider the complete decision context, rather than focus too narrowly on the quantitative measures of the prediction. Moreover, the research reports are not limited to the individual summary elements of earnings forecasts, such as whether to buy or sell a particular stock. Thus, it is evident that online security reports are a valuable reservoir of the reaction of stock market response to the message as feedback.

As noted above, there is still much room for performance improvement on stock movements forecasting. We have much work ahead of us. The predictive keyword vector extraction method we used is not sufficient to find the accuracy features in each document. The decision on keyword extraction is crucial because from an incorrect input nothing more than a meaningless output can be released. One of the shortcomings of our study is that only the historical stock index and semantics derived from the online research report are concerned. In the future, we will try to find and integrate more factors which can influence the stock market to develop a more accurate stock forecasting model.

In addition, the network construction procedure discussed in this paper is simple and straightforward. There are multiple ways of measuring the similarity among words, such as the log-likelihood ratio^45^, Chi-square, cosine and pointwise mutual information, etc. To the best of our knowledge, there is a lack of definitive evidence on the relative performance of different network construction procedures. Our empirical results show that the proposed SLS_L model performance can be improved significantly by incorporating network connected information. However, the adjacency measure is based on the Pearson correlation coefficient. This may limit our model in some context. It is possible that in practical data, adopting other network construction methods may further improve prediction and feature selection. Moreover, some preprocessing was performed on the nodes prior to the network construction and that significant "noise" may be removed. In future research, we will consider other ways of defining the similarity measure and adjacency matrix that can improve interpretability and reduce bias.

Lastly, how to apply our approach to practice is a challenging problem waiting to be explored. Guo et al.^46^, M’ng and Mehralizadeh^47^ have discussed the effectiveness of auto regression algorithms such as time series analysis. Given the market movement on a time-series, when the time-window is slid from the start to the end, the training and testing windows are captured sequentially. Future work will collect more volume of datasets containing mappings of text onto stock movements forecasting for multiple days that can explore the auto regression algorithm based on SLS_L for stock movement prediction. This strategy has the attractive property of effectiveness in the practical stock market analysis.
